# Toxicity of essential oil from Indian borage on the larvae of the African malaria vector mosquito, *Anopheles gambiae*

**DOI:** 10.1186/1756-3305-5-277

**Published:** 2012-12-03

**Authors:** Eliningaya J Kweka, Annadurai Senthilkumar, Venugopalan Venkatesalu

**Affiliations:** 1 Division of Livestock and Human Diseases Vector Control, Tropical Pesticides Research Institute, Ngaramtoni, Off Nairobi Road, P.O. Box 3024, Arusha, Tanzania; 2 Department of Botany, Annamalai University, Annamalai Nagar, Tamil Nadu, 608 002, India

## Abstract

**Background:**

Essential oils are currently studied for the control of different disease vectors, because of their efficacy on targeted organisms. In the present investigation, the larvicidal potential of essential oil extracted from Indian borage (*Plectranthus amboinicus*) was studied against the African anthropophagic malaria vector mosquito, *Anopheles gambiae.* The larvae of *An. gambiae* s.s laboratory colony and *An. gambiae* s.l of wild populations were assayed and the larval mortality was observed at 12, 24 and 48 h after exposure period with the concentrations of 3.125, 6.25, 12.5, 25, 50 and 100 ppm.

**Findings:**

Larval mortality rates of the essential oil was entirely time and dose dependent. The LC_50_ values of the laboratory colony were 98.56 (after 12h) 55.20 (after 24 h) and 32.41 ppm (after 48 h) and the LC_90_ values were 147.40 (after 12h), 99.09 (after 24 h) and 98.84 ppm (after 48 h). The LC_50_ and LC_90_ values of the wild population were 119.52, 179.85 (after 12h) 67.53, 107.60 (after 24 h) and 25.51, 111.17 ppm (after 48 h) respectively. The oil showed good larvicidal potential after 48 h of exposure period against *An. gambiae*. The essential oil of Indian borage is a renowned natural source of larvicides for the control of the African malaria vector mosquito, *An. gambiae*.

**Conclusion:**

The larvicidal efficacy shown by plant extracts against *An. gambiae* should be tested in semi field and small scale trials for effective compounds to supplement the existing larval control tools.

## Findings

### Background

Malaria is the most important parasitic disease that has created major public health problems in the tropical and sub-tropical regions, in spite of the decrease of malaria cases and vector populations. According to a global estimate from The World Health Organization (WHO), 216 million people who were infected with malaria parasites in 2010, (of which approximately 81%, or 174 million cases, were reported from Sub-Saharan African) resulted in 6, 55, 000 malaria deaths (about 91% were in Africa) [1]. Approximately 86% of malaria deaths globally were children under 5 years of age. Mosquitoes of the *Anopheles gambiae* Giles complex are the principal and most efficient malaria vectors in the world [2]. Among the *An. gambiae* complex members, *An. gambiae* s.s. plays a wide role in transmission of the most dangerous parasite, *Plasmodium falciparum* in Sub-Saharan countries [2].

The use of synthetic insecticides for the control of adult anopheline mosquitoes through indoor residual house spraying and insecticide impregnated materials have shown to decrease in efficacy over time in recent years in various parts of Africa [3]. This is due to the tolerance of adult mosquitoes against synthetic insecticides used for bed nets/indoor residual spray [1] and the widespread use of a single class of insecticide increases the risk that mosquitoes will develop resistance, which could rapidly lead to a major public health problem [4]. Targeting the mosquito larval stage by using non synthetic or alternative larvicides can be of significant impact as larvae cannot move from breeding sites [5]. In recent years, searching for new mosquito control agents from natural sources such as essential oil has gained recognition among researchers in countries with a renowned biodiversity and large numbers of plants/essential oils have been reported to possess larvicidal and repellent activity [6-8].

Indian borage *Plectranthus amboinicus* (Lour.) Spreng] belongs to the family Lamiaceae and it grows naturally and is distributed in tropical Africa and Asia. In the previous studies of the essential oil of *P. amboinicus* for its chemical composition and larvicidal activity against *An. stephensi*[9], the oil showed marked larvicidal potential with the LC_50_ values of the oil being 33.54 (after 12 h) and 28.37 ppm (after 24 h) against *An. stephensi*[9]. Based on the previous report, the current study evaluated the toxicity of this essential oil against the laboratory colony (*An. gambiae* s.s) and wild populations of the African malaria vector mosquito, *An. gambiae* s.l.

### Methods

#### Plant materials and extraction of essential oil

The fresh leaves of Indian borage were collected from Mangudi (11°20^′^35 N, 079°41^′^24 E), Cuddalore district of Tamil Nadu, India during April, 2010. The leaves were cut into small pieces and subjected to hydro distillation using a Clevenger type apparatus for 4 h. The essential oil was dried over anhydrous sodium sulphate and the purified essential oil was stored at +4°C for further use. The specimen voucher was deposited at the herbarium, Department of Botany, Annamalai University. The specimen voucher number is **AUBOT#231.**

#### Larval collection and rearing of mosquitoes

*An. gambiae s.s.* laboratory colony was reared in an insectary and maintained at 27.0 ± 2°C and relative humidity of 70 to 80%. The light regime was L 12: D 12. These females were provided with sugar solution (10% sucrose). Species identification of the *An. gambiae* s.s. was performed and confirmed by using polymerase chain reaction as described by the protocol of Scott *et al*. [10]. Eggs laid were collected on wet filter papers and kept in an incubator for 48 hours before hatching. Larvae were fed with diet of Brewer’s yeast until they developed to third instar larvae. Wild populations of larvae were sampled from natural habitats using a standard dipper of 350 ml in areas with a history of pyrethroid resistance observed in adult populations in western Kenya. Sampled larvae were sorted in an insectary and only third instar larvae of *An. gambiae* were used for larvicidal activity.

#### Mosquito larvicidal assay

The larvicidal activity was analysed as per the standard procedures recommended by the World Health Organisation [11]. The essential oil was dissolved in 1 ml of acetone and prepared in different concentrations viz., 3.125, 6.25, 12.5, 25, 50 and 100 ppm with distilled water. Twenty larvae (in a 20 ml beaker) of late third instar stage were used for larvicidal assay and five replicates were maintained for each concentration. Larval mortality was counted after 12, 24 and 48 h exposure and during the experimental period no food was offered to the larvae. All moribund mosquito larvae were considered as dead. The larval mortality was also checked for water and acetone individually. The lethal concentrations, LC_50_ and LC_90_ and their 95% confidence limit of upper and lower confidence levels were calculated by probit analysis in a Statistical package for social scientists, version 11.5 (SPSS Inc., Chicago, IL).

#### GC and GC-MS analysis

Gas chromatography (GC) analysis was carried out using Varian 3800 gas chromatography equipped with mass selective detector coupled to front injector type 1079. The chromatograph was fitted with DB 5 MS capillary column (30 m×0.25 mm i.d., film thickness 0.25 μm). The injector temperature was set at 280°C, and the oven temperature was initially at 45°C then programmed to 300°C at the rate of 10°C/min and finally held at 200°C for 5 min. Helium was used as a carrier gas with the flow rate of 1.0 ml/min. One microlitre of the sample (diluted with acetone 1:10) was injected in the split mode in the ratio of 1:100. The percentage of composition of the essential oil was calculated by the GC peak areas. GC–mass spectrometry (GC-MS) analysis of essential oil was performed using Varian 3800 gas chromatography equipped with Varian 1200L single quadrupole mass spectrometer. GC conditions were the same as reported for GC analysis and the same column was used. The mass spectrometer was operated in the electron impact mode at 70 eV. Ion source and transfer line temperature was kept at 250°C. The mass spectra were obtained by centroid scan of the mass range from 40 to 1,000 amu. The retention indices were calculated using a homologous series of n-alkanes, C_8_–C_22_ and n-octane was used as the internal standard. The compounds were identified based on the comparison of their retention indices (RI), retention time (RT) and mass spectra of WILEY, NIST library data of the GC-MS system and literature data [12].

### Results and discussion

The analysis of the chemical contents of the samples of essential oil from *P. amboinicus* revealed that the essential oil had 26 compounds (Table 1). The essential oil of Indian borage showed good larvicidal toxicity (Table 2) against both *An. gambiae* larvae of a laboratory colony and wild populations. The laboratory colony showed 100 per cent larval mortality at 100 ppm after 48 h of exposure period. The LC_50_ values of essential oil against the larvae of the laboratory colony was 98.56, 55.20 and 32.41 ppm and LC_90_ values were 14.40, 99.09 and 98.84 ppm after 12, 24 and 48 h of exposure period respectively. The larvae of the wild population showed poor response for the essential oil after 12 h of exposure period. Even though, the essential oil showed low LC_50_ value (25.51 ppm) after 48 h of exposure period. The essential oil showed the LC_50_ values of 119.52 (after 12 h) and 67.53 ppm (after 24 h). Acetone and water showed no mortality after 24 and 48 h of exposure period. The larval mortality rate of the essential oil was entirely time and dose dependent. The results of the present study are comparable to the previous study made by Senthilkumar and Venkatesalu [9]. The essential oil of *P. ambonicus* showed larvicidal activity against *An. stephensi* reared in the laboratory with the LC_50_ values of 33.54 (after 12 h) and 28.37 ppm (after 24 h). The larval mortality ability of the essential oil may be accredited by the presence of Carvacrol in the essential oil [13]. The results of the present study are also similar to that of earlier investigations on larvicidal activity of essential oils. Pavela and others reported that the essential oils obtained from *Thymus vulgaris, Satureja hortensis* and *Thymus satureioides* showed the highest larvicidal effect (LC_50_ 33, 36 and 44 μg/ml, respectively) after 24 h of exposure [14]. Stem distilled essential oil from *Zingiber officinalis* was evaluated for larvicidal activity against the filarial mosquito, *Culex quinquefasciatus*. The late third instar larvae showed the LC_50_ value of 50.78 ppm after 24 h of treatment [15]. It is apparent from the above mentioned results, the toxicity of essential oil against different mosquito species are not uncommon due to its chemical variations [16].

**Table 1:** **Chemical composition of the essential oil from the leaves of *P. amboinicus***[9]

**Peak No**	**AI**	**Chemical compound**	**Percentage**
1	788	1-octene	0.35
2	988	myrcene	0.16
3	1014	α-terpinene	0.61
4	1020	ρ-cymene	6.51
5	1025	β-phelandrene	0.11
6	1054	γ- terpinene	7.76
7	1098	trans-sabinene hydrate	0.22
8	1123	methyl octanoate	0.42
9	1165	borneol	0.26
10	1186	α-terpineol	3.28
11	1192	dihydro carvel	0.23
12	1195	methyl chavicol	0.28
13	1197	2Z-octenol acetate	0.96
14	1289	thymol	21.66
15	1298	carvacrol	29.25
16	1305	undecanal	8.29
17	1452	α-humulene	9.67
18	1489	β-selinene	2.01
19	1582	caryphyllene oxide	5.83
20	1584	2-phenyl ethyl tiglate	1.38
21	1590	β- copaen-4-α-ol	0.12
22	1608	humulene epoxide II	0.11
23	1611	tetradecanal	0.12
24	1615	β-himachalene oxide	0.15
25	1621	β-cedrene epoxide	0.03
26	1627	1-epi-cubenol	0.16
		**TOTAL**	**99.93**

*AI* (Arithmetic Index) in the literature [12].

**Table 2:** **Larvicidal activity of essential oil from Indian borage against a laboratory colony and wild population of African malarial vector mosquitoes, *Anopheles gambiae *s.s. after 12 and 24 hours 12, 24 and 48 h of exposure period respectively**

	**Concentration (ppm)**	**Time**	**Mortality**	**L C**_**50**_**(LCL-UCL)**^**a**^	**LC**_**90**_**(LCL-UCL)**^**a**^	***X***^**2**^**(df=4)**^**b**^
Laboratory colony (*An. gambiae* s.s)	3.125	After 12 Hours	0±0.0	98.56 (76.55 – 158.18)	147.40 (112.35 – 276.75)	25.52
	6.25		0±0.0			
	12.5		0±0.0			
	25		0±0.0			
	50		26±1.30			
	100		55±1.58			
	3.125	After 24 Hours	3±0.89	55.20 (35.26 – 96.69)	99.09 (70.65 – 203.33)	49.36
	6.25		5±0.70			
	12.5		14±0.83			
	25		16±1.48			
	50		83±1.67			
	100		98±0.54			
	3.125	After 48 Hours	23±1.14	32.41 (8.23 – 62.40)	98.84 (66.67 – 242.69)	30.57
	6.25		29±0.70			
	12.5		51±0.83			
	25		55±1.0			
	50		95±0.70			
	100		100±0.0			
Wild population (*An. gambiae* s.l)	3.125	After 12 Hours	0±0.0	119.52 (98.53 – 164.94)	179.85 (143.12 – 268.45)	8.19
	6.25		0±0.0			
	12.5		1±0.44			
	25		2±0.89			
	50		15±1.58			
	100		37±1.14			
	3.125	After 24 Hours	0±0.0	67.53 (55.31 – 85.22)	107.60 (88.92 – 143.21)	16.23
	6.25		0±0.0			
	12.5		3±0.89			
	25		14±0.83			
	50		47±1.14			
	100		96±0.83			
	3.125	After 48 Hours	42±1.67	25.51 (0.25 – 46.94)	111.17 (76.91 – 238.56)	16.63
	6.25		44±2.68			
	12.5		52±1.51			
	25		52±2.07			
	50		95±0.70			
	100		98±0.54			

^a^ – 95% Confidence interval.

^b^ – Degrees of freedom.

*LCL* Lower Confidence level, *UCL* Upper Confidence level.

Malarial transmission is much more difficult to control in Africa as compared to most other places because of a complex ecological system, it can be prevented by effective vector control targeting larval habitats. Otherwise, endemic malaria cannot be eliminated from most parts of Sub-Saharan Africa [17]. In that way, the essential oil of Indian borage is a renowned natural source of larvicides for the control of the African malaria vector mosquito, *An. gambiae*.

### Conclusion

The findings of this study have shown high mortality of *An. gambiae* s.l. third instar larvae caused by evaluated essential oils was dosage and time dependant. Further evaluations are ongoing for efficacy in semi field and subsequently small scale trials.

## Competing interests

Authors declare to have no competing interest in this work.

## Authors' contribution

EJK and VV conceived and designed the study. AS, VV and EJK carried out the data analysis and interpretation. EJK, VV and AS wrote the manuscript and approved the final version for submission. All authors read and approved the final manuscript.
